# Beliefs about Sexual Assault in India and Britain are Explained by Attitudes Toward Women and Hostile Sexism

**DOI:** 10.1007/s11199-017-0880-6

**Published:** 2018-01-06

**Authors:** Suzanne Hill, Tara C. Marshall

**Affiliations:** Division of Psychology, Department of Life Sciences, Brunel University London, Uxbridge, UB8 3PH UK

**Keywords:** Gender roles, Gender equality, Culture, Sex offences, Sexism, Rape

## Abstract

As many as one in five women worldwide will be sexually assaulted over the course of her lifetime (United Nations 2008), yet myths that downplay the prevalence and severity of sexual assault are still widely accepted. Are myths about sexual assault (rape myths) more likely to be accepted in cultures that endorse more traditional gender roles and attitudes toward women? To explore the relationships among rape myth acceptance, attitudes toward women, and hostile and benevolent sexism, data were collected from 112 Indian and 117 British adults, samples from two cultures differing widely in their gender role traditionalism. Analyses confirmed a cultural difference in rape myth acceptance, with the more traditional culture, India, accepting myths to a greater extent than the more egalitarian culture, Britain. Indian participants’ greater rape myth acceptance was explained by their more traditional gender role attitudes and hostile sexism. We discuss ways in which promoting gender egalitarianism may help to break down negative beliefs and reduce the stigma surrounding sexual assault, especially in India, for example through interventions which increase exposure to women in less traditional roles (e.g., those in positions of power).

*If my daughter or sister engaged in pre-marital activities and disgraced herself and allowed herself to lose face and character by doing such things, I would most certainly … put petrol on her and set her alight.* AP Singh, lawyer for those convicted of the rape and murder of India’s Daughter (BBC News, March 2015; Udwin 2015)

What is the role of culture in sustaining attitudes and beliefs about sexual assault? Due to the pervasive stigma surrounding sexual assault and subsequent low reporting rates, it is difficult to gain a complete understanding of the prevalence of sexual assault across cultures. According to a report from UN Women (Turquet et al. 2012), it is estimated that only 11% of sexual assaults are reported worldwide, although this varies by country. In India, this estimate drops to just 2% (Palermo et al. 2013), whereas in Britain, at 18%, the estimate is higher than the worldwide average (Myhill and Allen 2002). One factor that has promoted the stigma surrounding rape is the acceptance of *rape myths*, which are a set of attitudes and opinions suggesting victims are at fault for having been raped (Brownmiller 1975). Rape myth acceptance is influenced by a number of factors, including attitudes toward women (Costin and Schwarz 1987; Das et al. 2014), as well as hostile and benevolent sexism—that is, the rejection of women who defy traditional gender roles and the praising of women who abide by traditional gender roles, respectively (Chapleau et al. 2007; Glick and Fiske 1996). Traditional gender roles, and in the present article gender-traditional cultures (i.e., those with higher levels of country-level gender inequality: Schwartz and Rubel-Lifschitz 2009), refer to contexts in which roles are generally defined by biological sex, with men expected to provide income for the family whereas women look after children and maintain households (Read 2003). On the other hand, egalitarian gender roles, promoted by what we refer to here as egalitarian cultures, deem men and women to be equally capable of performing tasks within the home, family, community or workplace (Scott et al. 1996).

A number of studies have looked at the links between rape myth acceptance and perceptions of women in both Western (Burt 1980; Suarez and Gadalla 2010) and non-Western (Kanekar and Kolsawalla 1980; Lee et al. 2012) contexts. Gender differences have consistently been observed, with men reporting more traditional attitudes toward women and higher levels of sexism than women do (Olson et al. 2007; Zawisza et al. 2015), as well as more rape myth acceptance (Johnson et al. 1997). No previous study to our knowledge, however, has examined the relationships among rape myth acceptance, attitudes toward women, and hostile and benevolent sexism in a cross-cultural context, comparing a more egalitarian culture, Britain, with a more traditional culture, India. The widely-publicised gang rape and murder of 23-year-old Jyoti Singh, who came to be known as “India’s Daughter,” in Delhi, December 2012 (Chaudhuri and Fitzgerald 2015) illustrates the importance of considering how beliefs about sexual assault function in Indian society. The low reporting rates in India, and the higher rates in Britain, make these cultures ideal for comparison because beliefs about sexual assault in Britain may differ from India in ways that could help to explain the lack of reporting of sexual offences.

## Beliefs about Sexual Assault

Rape myths are stereotypical beliefs about sexual assault, which tend to blame victims rather than perpetrators, and trivialise the violence behind sexual assault (Brownmiller 1975). Rape myth acceptance and victim-blaming beliefs have been studied extensively in Western cultures, but less research has been conducted in non-Western contexts. Because sexual assault is prevalent worldwide (Jewkes et al. 2013), it is important that studies take into consideration cultural differences in factors related to rape (e.g., beliefs about sexual assault) and how these can impact outcomes (e.g., reporting rates).

In one of the earliest studies of rape myths, Burt (1980) found that attitude variables, such as acceptance of interpersonal violence, and demographic variables, such as age and occupational status, were associated with rape myth acceptance in the United States. Costin (1985) also found in a U.S. sample that beliefs reflecting negative stereotypes about rape were positively related to beliefs about the restriction of women’s roles. One strength of his study is that it used both student and nonstudent samples and therefore could be widely generalized across populations, although cross-cultural applicability beyond American samples is questionable. In 1987, however, Costin and Schwarz (1987) conducted a similar study with participants from the United States, England, Israel, and West Germany, and they were able to replicate the previous findings, thus providing some evidence of the existence of rape myths across several Western cultures. Many of the findings of this early research have been observed in the twenty-first century as well (see Grubb and Harrower 2008, and Grubb and Turner 2012, for reviews), suggesting that the need for further research on rape myths is not an outdated issue.

Researchers have also found evidence of rape myths and rape myth acceptance across a wider range of cultures. A body of research by Kanekar and Kolsawalla (1977, 1980, 1981) has shown that rape victim-blaming is prevalent in India, with male participants attributing greater responsibility to victims and sympathizing more with rapists than female participants do. However, Kanekar and Kolsawalla’s research was conducted using university student samples, making applicability to the general Indian population difficult. Because education to degree level is related to more liberal attitudes (Phinney and Flores 2002), it is possible that the Indian population as a whole has even higher levels of rape victim-blaming than were observed in Kanekar and Kolsawalla’s studies. As with the studies we cited, this body of work is over 30 years old, and in that time, Indian society has undergone large changes (Corbridge et al. 2013), suggesting the importance of further research into whether these findings are still observed today. In more recent research, Kanekar (2007) goes on to suggest that, in India compared to America, victims of rape and sexual assault are treated more harshly by society. This suggestion is supported by Nayak et al. (2003) who found that American students were more positive, or less negative, about sexual assault victims than were Indian students.

In addition to the existence of rape myths across cultures, other research has looked at the degree to which individuals accept rape myths and whether this differs cross-culturally. Mori et al. (1995) found that Asian American college students had more negative attitudes both toward rape victims and toward women, as well as accepted more rape myths, than did White American students. Although this study was conducted in the United States with an Asian American sample, it highlights important cultural differences that could influence rape myth acceptance such as the Asian emphasis on harmony and the importance of avoiding impropriety and shame (Mori et al. 1995). Ward et al. (1988) looked at attitudes toward rape victims, or rape myth acceptance, across cultures and found that 14 countries, including India and Britain, all accepted rape myths to some degree. Their findings also show that Indian participants had higher levels of rape myth acceptance than did British participants. Although attitudes toward women have become less traditional since many of these studies were published 30–40 years ago (Bolzendahl and Myers 2004), these attitudes still remain more traditional in India compared to the United Kingdom (Brandt 2011; Ward 1988), suggesting that we may observe a corresponding cultural difference in rape myth acceptance.

## Attitudes Toward Women

Cultural differences in attitudes toward women may help to explain cultural differences in rape myth acceptance. Attitudes toward women, gender equality, and the reduction of gender role stereotyping have always been central to women’s rights movements, which occur worldwide though often take culture-specific forms (Baird and Obaid-Chinoy 2004; Crawford 2003). Gender roles have been defined as expectations applied to individuals on the basis of their biological sex, and attitudes toward women, or gender role ideology, as individual views of appropriate roles for men and women (Boehnke 2011). Although men typically report more traditional views toward gender roles than women do (Olson et al. 2007), most industrialized countries have shifted toward more egalitarian gender roles and gender role attitudes within the last 50 years (Boehnke 2011). Bolzendahl and Myers (2004) found that attitudes toward women liberalised in the United States between 1974 and 1998, especially in terms of sexual behaviour, public sphere gender roles, and family responsibilities.

Research that has focused on the links between attitudes toward women and beliefs about sexual assault has consistently found that traditional attitudes toward women are related to more rape myth acceptance (Lutz-Zois et al. 2015). Costin (1985); Costin and Schwarz 1987) found that traditional attitudes toward women were positively correlated with negative attitudes toward rape victims and increased rape myth acceptance. Research by Das et al. (2014), on adolescent boys in India, found that those who had inequitable, or traditional, gender role attitudes tended to be more likely to condone sexual violence.

## Sexism

In addition to attitudes toward women, sexism may also influence beliefs about sexual assault. Ambivalent sexism is comprised of two constructs: hostile sexism and benevolent sexism. Hostile sexism refers to the rejection of women who defy traditional gender roles whereas benevolent sexism refers to rewarding women who abide by traditional gender roles (Glick and Fiske 1996). To study these types of sexism, Glick and Fiske (1996) developed the Ambivalent Sexism Inventory (ASI). This scale measuring hostile and benevolent sexism is not only applicable across genders (Glick and Fiske 1996), but also across cultures. Glick et al. (2000) tested the ASI on 15,000 men and women in 19 different nations, including England but not India, and they found that hostile and benevolent sexism were positively correlated across cultures. They also found that the national average score on benevolent and hostile sexism scales predicted gender inequality for all cultures studied. Another large-scale study by Brandt (2011), based on data from 57 countries, including Britain and India, found that, in general, men had higher levels of sexism than women did and that sexism directly predicted increased gender inequality.

Many oppressive belief systems, such as racism, classism, homophobia, and sexism, are linked to the acceptance of rape myths (Aosved and Long 2006). A meta-analysis of 37 studies conducted in North America found hostile attitudes and behaviours toward women, as well as other prejudices such as racism, classism and ageism (Suarez and Gadalla 2010), were consistently associated with rape myth acceptance. Other research, primarily conducted in Western contexts, has found links between sexism and rape myth acceptance (Viki and Abrams 2002). Abrams et al. (2003) found that higher scores on both benevolent and hostile sexism scales were linked to more victim-blaming and stronger acceptance of rape myths. They also found that, for male participants, higher hostile sexism was related to higher rape proclivity (i.e., a predisposition toward sexual harassment or committing rape; Begany and Milburn 2002). The studies by Abrams et al. were all conducted on British samples, as was a study by Pedersen and Strömwall (2013) who found that British participants attributed higher levels of blame to victims than did Swedish participants and that this was positively related to their level of benevolent sexism. Because there is generally more gender equality in Sweden than in Britain, and more in Britain than in India (Brandt 2011), it is reasonable to expect that a British sample would have lower levels of sexism and victim-blaming than an Indian sample would.

## Gender Differences

Notwithstanding a few studies which have found no gender differences in attitudes relating to gender and rape myth acceptance (Acock and Ireland 1983; Sims et al. 2007), others have generally shown that men have more negative (e.g., hostile or derogatory), stereotypical, and traditional views than women do (Costin and Schwarz 1987; Kanekar and Kolsawalla 1980; Zawisza et al. 2015). There are a number of reasons why men tend to have more traditional views than women do; for example, patriarchal societal structures are more beneficial to men than to women (e.g., across cultures women are paid less than men; UN Women 2015), and therefore men may be more inclined to hold beliefs that sustain their privilege. Similarly, because beliefs and attitudes tend to be self-serving, women are more likely to hold egalitarian beliefs about their own roles because traditional gender roles are often restrictive and limit women’s access to employment and education (Röder 2014).

A British study by Davies et al. (2012) found that male participants had higher rape myth acceptance, more stereotypical gender views, and higher levels of hostile sexism than did female participants. This gender difference has also been found in India, with more responsibility being assigned to victims by male participants than by female participants (Kanekar and Kolsawalla 1977). These gender differences may stem from the different functions rape myth acceptance fulfils for each gender. Men typically use rape myths to justify male sexual violence whereas women use them to deny their own personal vulnerability (Lonsway and Fitzgerald 1995). Based on these findings, we expected in the present study that men in Britain and in India, relative to women, will report more traditional attitudes toward women, have higher levels of hostile and benevolent sexism, and accept rape myths to a greater extent.

## The Present Study

The present study investigated three factors that previously have been identified as predictors of rape myth acceptance: attitudes toward women and hostile and benevolent sexism. Previous research has looked at links among culture, attitudes toward women or sexism, and rape myth acceptance, but ours is the first known study to examine these variables in a single mediating model. By collecting data from British and Indian samples, we aimed to facilitate an understanding of potential reasons behind differences in rape myth acceptance and, by extension, the different rates of reporting sexual assault in the two countries. Our study also aimed to fill gaps left by previous research that focused primarily on North American samples. Thus we propose the following hypotheses hypotheses. (a) There will be a main effect of gender, such that men from both cultures will have more traditional attitudes toward women, be higher in hostile and benevolent sexism, and accept rape myths more than will women in both cultures (Hypothesis 1). (b) There will be a main effect of culture, with Indian participants reporting more traditional attitudes toward women, higher levels of hostile and benevolent sexism, and higher levels of rape myth acceptance than British participants will (Hypothesis 2). (c) The cultural difference in rape myth acceptance will be mediated by attitudes toward women, hostile sexism, and benevolent sexism. Thus, compared to British participants, Indian participants will report more traditional attitudes toward women and more hostile and benevolent sexism; in turn, they will report greater rape myth acceptance (Hypothesis 3).

## Method

### Participants

The total number of participants was 229, with 112 (48.9%) Indian and 117 (51.1%) British participants. The mean age of Indian participants was 32.23 (*SD* = 8.70, range = 21–69); 35 (31.3%) were female, and 77 (68.8%) were male. The mean age of British participants was 32.07 (*SD* = 15.02, range = 18–73); 75 (64.1%) were female, 40 (34.2%) were male, and 1 (.9%) was genderfluid. Fully 110 (98%) Indian participants reported being educated to degree level or above, compared to 69 (59%) British participants; 91 (81%) Indian participants were in employment, compared to 65 (56%) British participants (38, 33%, of the British sample were students). Majorities—83 (74%) Indian and 74 (63%) British participants—were in a relationship, cohabiting and/or married. Age was not significantly different between Indian and British participants (*p* = .96), however both gender, χ^2^(1) = 26.21, *p* < .001, Cramer’s *V* = .34] and education (proportion educated to degree level), χ^2^(1) = 49.48, *p* < .001, Cramer’s *V* = .47, were significantly different between groups, and therefore they have been included as controls in the analysis for Hypothesis 2. Education is also included as a control for Hypothesis 1.

### Materials

Questions were presented in the same order to all participants: demographic questions, Attitudes toward Women scale, Attitudes toward Rape Victims scale, and Ambivalent Sexism Inventory. All participants completed the questionnaire in English because they were either from Britain or India, both countries where English is an official language.

#### Attitudes toward Women Scale

Spence et al.’s (1973) Attitudes toward Women Scale (short version) contains 25 items arranged into two subscales: traditional attitudes and egalitarian attitudes. An example item from the traditional subscale is “Intoxication among women is worse than intoxication among men,” and an example item from the egalitarian scale is “A woman should be as free as a man to propose marriage.” Participants were asked to rate items on a 7-point Likert scale from 1 (*Strongly Disagree*) to 7 (*Strongly Agree*). Scores on items from the egalitarian subscale were reverse-coded and used alongside scores from the traditional subscale to create a summed attitude score, wherein higher scores reflected more traditional attitudes toward women. The Cronbach’s alpha for the scale was .82 for the British sample and .87 for the Indian sample.

#### Attitudes toward Rape Victims Scale

Ward’s (1988) Attitudes toward Rape Victims Scale, consisting of 25 items, measures positive and negative attitudes toward rape victims and thus whether or not the respondent accepts rape myths and victim-blaming. Examples of positive items on the scale include “Women do not provoke rape by their appearance or behaviour” and “A woman should not blame herself for rape,” whereas examples of negative items include “Women often claim rape to protect their reputations” and “In most cases when a woman was raped she deserved it.” Participants were asked to rate each item on a 7-point Likert scale from 1 (*Strongly Disagree*) to 7 (*Strongly Agree*). Scores for positive items were reverse-coded and summed with scores for negative items such that higher scores reflect more negative attitudes toward rape victims and thus increased victim-blaming and acceptance of rape myths. Due to the sensitive nature of this scale, an additional consent question was added before participants reached the items, which informed participants that the scale was potentially triggering or upsetting and asked them to give consent to answer the questions. Participants who did not give their consent were redirected to the next set of questions. Significantly more Indian participants (*n* = 16, 9 female) than British (*n* = 0) answered “No” to the additional consent question prefacing the Attitudes toward Rape Victims scale, χ^2^ (1, 229) = 17.97, *p* < .001. Cronbach’s alphas for the scale were .82 for the British and .87 for the Indian sample.

#### Ambivalent Sexism Inventory

The Ambivalent Sexism Inventory (Glick and Fiske 1996) is designed to measure two components of sexism: hostile sexism and benevolent sexism. The subscales reflecting hostile and benevolent sexism each contain 11 items. An example of a hostile sexism item is “Most women interpret innocent remarks as sexist,” and an example of a benevolent sexism item is “Men should sacrifice to provide for women.” Participants were asked to rate each item on a 7-point Likert scale from 1 (*Strongly Disagree*) to 7 (*Strongly Agree*). The Cronbach’s alpha for the hostile sexism subscale was .90 for the British sample and .71 for the Indian sample. The Cronbach’s alpha for the benevolent sexism subscale was .86 for the British sample and .67 for the Indian sample. Previous research by Glick and colleagues also found differences in alphas across cultures; however, they did not believe this to be an issue given the good model fit observed (Glick et al. 2000).

In line with Glick and Fiske’s (1996) tripartite model of benevolent sexism, we factor analysed items from the benevolent sexism scale; however, the three-factor model was only observed in the UK sample. We also examined the reliability analysis for each subscale of benevolent sexism. For Protective Paternalism, Cronbach’s alpha was .69 for the British sample and .38 for the Indian sample. For Complementary Gender Differentiation, Cronbach’s alpha was .81 for the British sample and .77 for the Indian sample. For Heterosexual Intimacy, Cronbach’s alpha was .75 for the British sample and .39 for the Indian sample. Because the three-factor model did not emerge in the Indian data, and the alphas for two of the three subscales were very low in the Indian sample, we decided to use total scores for benevolent sexism, which was more reliable.

### Procedure

A majority of Indian participants were recruited using Amazon’s Mechanical Turk (MTurk), and they were paid $ .50 USD for completing the questionnaire. British participants were primarily recruited through social media, a Research Participation page on the Principal Investigator’s university intranet, and via word of mouth. They were given the opportunity to enter a prize draw to win one of four £20 Amazon vouchers as an incentive for participation. Prolific Academic (www.prolific.ac) was also used to recruit two British participants, who were paid £1.25 for their participation. The recruitment methods vary by country due to feasibility of data collection. That MTurk is a useful tool for collecting data from Indian participants has been recognised by a number of researchers (Bejanyan et al. 2014; Elischberger et al. 2017), and at the time of data collection, MTurk was not available for recruiting British participants, thus other methods were used. Although incentives differed across recruitment methods, the opportunity for remuneration was offered to all participants.

## Results

### Hypothesis 1: Gender Differences

Descriptive statistics and correlations for all scales are reported in Table 1. Correlations between all variables were significant within the British sample, but this was not the case for the Indian sample, for whom benevolent sexism was not significantly correlated with both hostile sexism and with rape attitudes.

**Table 1:** Descriptive statistics and correlations among study variables

	Indian sample				British sample				Correlations			
	Men	Women	Total		Men	Women	Total					
Variables	*M* (*SD*)	*M* (*SD*)	*M* (*SD*)	*n*	*M* (*SD*)	*M* (*SD*)	*M* (*SD*)	*n*	1	2	3	4
1. Attitudes toward women	92.86 (17.50)	88.94 (21.98)	91.62 (19.01)	111	49.84 (11.64)	43.79 (13.30)	45.67 (13.06)	103	–	61**	.01	.82**
2. Hostile sexism	47.36 (8.60)	46.86 (9.16)	47.20 (8.75)	111	32.50 (13.75)	27.21 (14.50)	29.00 (12.49)	107	.66**	–	.28**	.63**
3. Benevolent sexism	49.71 (8.16)	51.94 (9.01)	50.41 (8.46)	111	39.05 (10.66)	29.99 (10.97)	33.09 (11.65)	108	.58**	.47**	–	.06
4. Attitudes toward rape victims	93.38 (22.05)	89.87 (26.41)	92.38 (23.26)	81	55.44 (18.86)	45.00 (12.72)	48.48 (15.75)	102	.56**	.73**	.43**	–

Correlations for the Indian sample are presented above the diagonal; for the British sample, below

** *p* < .01

The first hypothesis, that men from both cultures will have more traditional attitudes toward women, be higher in hostile and benevolent sexism, and accept rape myths more than women will in both cultures, was not supported by the data. A MANCOVA was conducted to explore the main and interaction effects of gender and culture, but results for the main effect of gender were not significant (*V* = .03), *F*(4165) = 1.20, *p* = .311. There was a significant interaction between gender and culture (*V* = .07), *F*(4165) = 3.26, *p* = .013, ηp^2^ = .07, and subsequent ANOVA analysis (using a Bonferroni-corrected alpha of .0125) found that this interaction effect was evident for only one scale, benevolent sexism, *F*(1219) = 15.49, *p* < .001, ηp^2^ = .04,. Simple effects testing revealed that British men were significantly higher in benevolent sexism than were British women *p* < .001, *d* = .41, but there was no significant difference between Indian men and women, *p* = .230. Means for all groups and scales are available in Table 1.

### Hypothesis 2: Cultural Differences

Hypothesis 2—that Indian participants will be more likely to accept rape myths than will British participants—was supported by the data. The results of the previous MANCOVA analysis showed a significant effect of culture across measures (*V* = .59), *F*(4165) = 59.90, *p* < .001, ηp^2^ = .59), and a series of ANOVAs were used to unpack this finding. Using a Bonferroni-corrected alpha of .0125, results showed significant main effects of culture across all scales: attitudes toward women, *F*(1214) = 3.85, *p* < .001, *d* = 1.74; hostile sexism, *F*(1218) = 111.26, *p* < .001, *d* = 1.28); benevolent sexism, *F*(1219) = 116.24, *p* < .001, *d* = 1.25; and attitudes toward rape victims, *F*(1182) = 162.81, *p* < .001, *d* = 1.57.

### Hypothesis 3: Mediation Model

A mediation model was used to test Hypothesis 3, using the PROCESS (Hayes 2013) macro for SPSS, with attitudes toward rape victims as the dependent variable, culture as the predictor (Britain = 1, India = 0), hostile sexism, benevolent sexism, and attitudes toward women as mediators, and gender (Male = 1, Female = 0) and education (No degree = 0, Degree = 1) as control variables The significance of indirect effects was tested using bootstrapping procedures, with 1000 bootstrap samples and 95% confidence intervals. Significant indirect effects did not include zero in the confidence interval. The indirect effect of culture on acceptance of rape myths was significant through attitudes toward women (*b* = −27.77, *SE* = 3.63, 95% CI [−34.81, −20.98]) and hostile sexism (*b* = −9.58, *SE* = 2.50, 95% CI [−14.54, −4.89]). The indirect effect of culture on acceptance of rape myths through benevolent sexism was not significant. (A moderated mediation was also conducted to test whether gender moderated the indirect effects of culture on rape myth acceptance through the three mediators; however, it was not significant, that is, confidence intervals for indirect effects contained zero, likely due to the lack of gender differences in the sample.)

## Discussion

The present study found that British and Indian participants significantly differed in their attitudes toward women, hostile and benevolent sexism, and rape myth acceptance. Moreover, Indians’ greater rape myth acceptance was mediated by their more traditional attitudes toward women and hostile sexism. This previously untested mediational model contributes to our theoretical understanding of the cultural factors involved in beliefs about sexual assault. We discuss these findings in more detail in the following.

### Gender Differences

The first hypothesis, that men from both cultures would have more traditional attitudes toward women, higher levels of hostile and benevolent sexism, and accept rape myths more than would women, was not supported by the data. This is inconsistent with much of the previous literature in the area, which has found that men have more negative attitudes toward rape victims (Abrams et al. 2003) and are higher in both hostile (Brandt 2011) and benevolent (Zawisza et al. 2015) sexism. It is possible, however, that gender differences would have been detected with a larger sample and greater statistical power.

Despite no overall gender differences, our results revealed a culture x gender interaction in the British sample, with British men scoring significantly higher on benevolent sexism than British women did. It is somewhat surprising that this gender difference was only significant in the British sample because female participants are consistently found to have lower levels of both hostile (Brandt 2011) and benevolent (Zawisza et al. 2015) sexism than male participants across cultures. However, other research has suggested that more egalitarian cultures such as Britain can sometimes have more marked gender differences than traditional cultures such as India (Schmitt et al. 2016).

### Culture Differences

The second hypothesis was largely supported: there was a clear cultural difference on all variables measured, and Indian participants’ greater acceptance of rape myths was mediated by their more traditional attitudes toward women and higher levels of hostile sexism. Benevolent sexism, however, was not a significant mediator. As predicted, rape myth acceptance was higher in Indian participants than in British participants, and this effect remained, albeit reduced, once hostile sexism and attitudes toward women were added into the mediation model. Benevolent sexism did not mediate the relationship between culture and rape myth acceptance, suggesting that hostile sexism played a larger role in attitudes surrounding sexual assault. This is not entirely surprising because both hostile sexism and rape myth acceptance tap negative attitudes toward women; benevolent sexism, on the other hand, taps into attitudes that could be viewed as superficially positive toward women (Glick and Fiske 1996).

These results confirm the findings of a number of previous studies, which have also observed links between beliefs about sexual assault, attitudes toward women, and sexism, although not in a single mediational model. Previous research found that traditional attitudes toward women and rape myth acceptance are related in both Western (Costin and Schwarz 1987; Lutz-Zois et al. 2015) and non-Western cultures (Das et al. 2014; Kanekar and Kolsawalla 1980) and that hostile sexism is also positively associated with rape myth acceptance (Chapleau et al. 2007; Suarez and Gadalla 2010). The present study not only replicated these previous findings, but also linked the variables (i.e., attitudes toward women, hostile sexism, and rape myth acceptance) together into one model.

In the present study, participants were asked an additional consent question just prior to the completion of the Attitudes toward Rape Victims scale; it gave participants the opportunity to choose not to answer these questions and instead move on to the next section. Of the small number of participants who declined to complete this scale, all were Indian, suggesting that they may be aware of higher levels of rape myth acceptance observed in India, perhaps due to media stories about India’s Daughter bringing negative beliefs about sexual assault in India to public attention (Lodhia 2015).

### Limitations and Further Research Directions

The strength of the present study is that it fills a gap in the literature on beliefs about sexual assault across cultures using both student and nonstudent samples, making it generalisable to a wider population. A limitation, however, is that participants may have been aware of the goals of our study, or at least were likely to be aware of socially desirable beliefs about sexual assault or perceptions of women. It is therefore possible that their responses to the measures were not a true reflection of their attitudes. This may partially explain why some Indian participants declined to answer the sensitive Attitudes toward Rape Victims scale. Participants may also have been aware that it is generally not socially acceptable to endorse gender-traditional or sexist attitudes toward women. A recommendation for further research, therefore, would be to include an impression management or social desirability scale such as the Marlowe-Crowne Social Desirability Scale (Crowne and Marlowe 1960), which has been successfully used across cultures (Mukherjee 1967).

Another limitation of our study is the varied range of recruitment strategies used in Britain and India. It was more feasible to recruit British participants than Indian students through convenience sampling because the researchers could easily access British participants through their own, and their university’s, network, whereas Indian participants were less easy to reach. Future research could endeavour to overcome this issue by recruiting all participants via MTurk or an alternative crowd-sourcing website such as Prolific Academic. Despite significant differences between the two groups in education level and gender ratios, both groups reported high levels of education and employment, suggesting at least somewhat comparable levels of socioeconomic status. As previously noted, the Indian sample recruited in our study was highly educated, and previous research has shown that those with a university education are likely to have more liberal values and attitudes than those without (Phinney and Flores 2002). Thus, the large effects of culture observed here might actually be conservative; they could be even larger in a more diverse sample.

The present study did not measure attitudes toward men and/or perpetrators of assault, choosing instead to focus on attitudes toward women. Although we recognize that men are also subject to domestic violence and sexual assault, the prevalence of gendered violence toward women is significantly higher (Jewkes et al. 2002), hence our choice to focus on women. Future research, however, could include measures of male role attitudes (e.g., the Ambivalence toward Men Inventory; Glick and Fiske 1999) to assess whether there are also cultural differences in attitudes toward men and male privilege (measured as resentment of paternalism) as well as what part they may play in rape myth acceptance.

### Practice Implications

Our findings also have practical implications: for one, they suggest that fear of repercussions may dissuade women from reporting sexual violence in India because they may face negative attitudes and be treated harshly when they do (Kanekar 2007). Second, our results highlight the need for interventions that may reduce negative perceptions of women and beliefs about sexual assault. By gaining a clearer understanding of the variables that are associated with negative beliefs about sexual assault (i.e., traditional attitudes toward women and sexism), it will become easier to design interventions that promote women’s rights and egalitarian attitudes, possibly leading to more positive (i.e., less victim-blaming) attitudes about sexual assault in both Indian and British populations. The link between traditional gender role attitudes and increased rape myth acceptance suggests that a potential intervention could be based around breaking down traditional gender roles and increasing exposure to egalitarian roles. This idea is supported by recent research by Taschler and West (2017) who found that contact with counter-stereotypical women (i.e., those in positions of power or authority) was related to lower sexism and decreased rape myth acceptance.

### Conclusion

The present study highlighted significant cross-cultural differences in beliefs about sexual assault and perceptions of women. Indian participants’ greater rape myth acceptance relative to British participants might help to explain the lack of reporting of sexual violence in India. In addition, Indians’ more traditional attitudes toward women and hostile sexism explained their greater rape myth acceptance.

There is a dearth of research looking at sexual assault and perceptions of women across cultures, and most Western research has focussed on North American samples rather than British or European samples (Chapleau et al. 2007; Costin and Schwarz 1987). In light of recent events in India that have brought sexual violence to the forefront of public attention, it is important to gain a clearer understanding of the function of negative beliefs about sexual assault in Indian society and other variables that are associated with these negative beliefs to suggest ways in which these beliefs can be broken down. By comparing the relationships between these beliefs in both British and Indian populations, we sought to identify aspects of British cultural values which may facilitate more positive attitudes toward women and, by extension, suggest potential interventions for reducing rape myth acceptance in both Britain and in India. Although reducing negative beliefs may not directly reduce crime, despite previous research has shown links between attitudes and rape proclivity (Abrams et al. 2003; Begany and Milburn 2002), it is feasible that a reduction in victim-blaming could increase reporting of incidents and subsequent prosecution of perpetrators, which in turn may deter individuals from committing such offences. Overall, our findings could be helpful in increasing understanding of the cultural factors that drive beliefs about sexual assault, thus enabling the development of interventions aimed at reducing their prevalence across cultures. This knowledge could subsequently reduce stigma and enable more people to feel able to report sexual assaults when they occur, hopefully reducing the prevalence of sexual assault and such cases as India’s Daughter (Chaudhuri and Fitzgerald 2015).
